# The effectiveness of guideline implementation strategies in the dental setting: a systematic review

**DOI:** 10.1186/s13012-019-0954-7

**Published:** 2019-12-17

**Authors:** Amy R. Villarosa, Della Maneze, Lucie M. Ramjan, Ravi Srinivas, Michelle Camilleri, Ajesh George

**Affiliations:** 1 Centre for Oral Health Outcomes and Research Translation (COHORT), Liverpool, 1871 Australia; 2 Western Sydney University, Penrith, 2751 Australia; 3 South Western Sydney Local Health District, Locked Bag 7103, Liverpool BC, NSW 1871 Australia; 4 Ingham Institute for Applied Medical Research, Liverpool, 1871 Australia; 5 Translational Health Research Institute, Western Sydney University, Penrith, 2751 Australia; 6 University of Sydney, Camperdown, 2050 Australia

**Keywords:** Dentistry, Implementation, Clinical guidelines

## Abstract

**Background:**

Guideline implementation has been an ongoing challenge in the dental practice setting. Despite this, there are no reviews summarising the existing evidence regarding effective guideline implementation strategies in this setting. In order to address this, this systematic review examines the effectiveness of guideline implementation strategies in the dental practice setting.

**Methods:**

A systematic search was undertaken according to the PRISMA statement across nine electronic databases, targeting randomised controlled trials and quasi-experimental studies which evaluated the effectiveness of guideline implementation strategies in improving guideline adherence in the dental setting. All records were independently examined for relevance and appraised for study quality by two authors, with consensus achieved by a third author. Data were extracted from included studies using a standardised data extraction pro forma.

**Results:**

A total of 15 records were eligible for inclusion in this review, which focused on the effects of audit and feedback, reminders, education, patient-mediated interventions, pay for performance and multifaceted interventions. Although there were some conflicting evidence, studies within each category of implementation strategy indicated a positive effect on guideline adherence.

**Conclusions:**

This study has identified education, reminders and multifaceted interventions as effective implementation strategies for the dental practice setting. Although this is similar to research findings from other health sectors, there is some evidence to suggest patient-mediated interventions may be less effective and pay for performance may be more effective in the dental setting. These findings can inform policy makers, professional associations, colleges and organisations in the future adoption of clinical guidelines in the dental practice setting.

**Trial registration:**

This systematic review was registered with the International Prospective Register of Systematic Reviews (PROSPERO), registration ID CRD42018093023.

Contributions to the literature

- Research summarising the effectiveness of guideline implementation strategies remains inconclusive and lacks focus on the dental setting, which has some of the lowest rates of guideline adherence.
- Similar to findings in other settings, audit and feedback, reminders, education, patient-mediated interventions and multifaceted interventions may be effective in the dental setting, and this study identified pay for performance as an additional effective strategy.
- These findings contribute to a recognised gap in the literature, by highlighting which implementation strategies may be the most effective for dental practitioners, which can serve to inform the future adoption of clinical guidelines in this sector.

## Background

With a growing emphasis on evidence-based practice in the clinical setting, health services are developing increasing numbers of clinical guidelines to direct more efficient clinical practice. Defined as “systematically developed statements to assist practitioner and patient decisions about appropriate health care for specific clinical circumstances”, clinical guidelines delineate appropriate and inappropriate care [1]. The overall goal of developing clinical guidelines is to improve the quality of care provided to patients by increasing clinical efficiency reducing inappropriate practices, thereby improving patient outcomes [2, 3]. They can also be a resource to inform patients regarding their clinicians’ decisions, and drive public policy by drawing attention to areas of need [4]. In addition, clinical guidelines can afford benefits to health care professionals as they can improve the quality of clinical decisions, support quality improvement activities and highlight gaps in the evidence, thus encouraging further research [4].

Despite the growing number of guidelines, the success of their use to change or introduce evidence-based health practices, otherwise known as implementation, is variable [5]. It has been acknowledged that the mere existence of clinical guidelines will not necessarily result in their implementation [6]. Research identifying determinants of guideline implementation across various settings has been extensively undertaken across the globe and has highlighted a myriad of factors that can impact the successful use of clinical guidelines [7–12]. These include individual health professional factors such as knowledge, awareness, self-efficacy, expectancy of positive outcomes, attitudes and intention [2, 7–14], as well as patient factors, such as applicability of guidelines to patients, patient preferences and behaviour [7, 8, 11]. Other factors such as organisational and environmental factors, as well as guideline complexity, can also potentially impact a clinician’s decision or ability to adhere to clinical guidelines [7–12, 14]. Therefore, guideline implementation strategies seek to address these potential barriers to compliance, and facilitate the application of new guidelines into practice [15].

Although research has focussed on evaluating which implementation strategies are the most effective in changing practice [16, 17], the evidence from these reviews were inconclusive, and often included studies that were most common in acute care or general practice settings [16, 17]. It has been acknowledged that these findings may not be applicable to other settings, such as the dental setting, which research suggests could be one of the clinical areas with lowest guideline compliance [18]. The dental setting has unique contextual factors that may impact the uptake of new guidelines, and these need to be considered when developing guideline implementation strategies [17, 19–21]. For example, the size of dental practices is highly variable, with some practitioners operating alone, and other practices involving more than 10 practitioners. This may have an influence on the success of guideline implementation, with evidence suggesting larger practices are more likely to comply with clinical guidelines [19]. With the push for dental practitioners to expand their scope of practice to include addressing health issues such as tobacco cessation [22], diabetes [23] and childhood obesity [24], it is essential to identify effective guideline implementation strategies specifically in the dental setting. With no studies synthesising the available findings in this area, the aim of this systematic review was to explore the effectiveness of various guideline implementation strategies in improving dental practitioners’ adherence to any clinical guidelines. This review specifically focussed on identifying changes in dental practitioners’ adherence, that is behaviour change, to any clinical guidelines they may have to follow, both dental and non-dental.

## Methods

### Research design

A systematic review was conducted according to the framework developed by Khan et al. [25] and reported according to the preferred reporting items for systematic reviews and meta-analyses (PRISMA) statement (Additional file 1) [26]. This approach was chosen as it appropriately addressed the study aims to systematically synthesise the existing evidence regarding the effectiveness of each type of implementation strategy. Several study authors are experienced in systematic review methodologies, having published multiple systematic reviews across various fields [27–30].

### Searches

Databases including Scopus, CINAHL, MEDLINE, ProQuest, Embase, Cochrane, PsycINFO and Web of Science, as well as Google Scholar, were extensively searched from October 2018 to April 2019 (Additional file 2). With the assistance of a librarian, a combination of Boolean operators, truncations and Medical Subject Headings (MeSH) were used to develop individualised search strategies according to the indexing terms of each database. These search strategies incorporated key words such as *guideline, recommendation, consensus, implementation, dissemination, translation, strategy, approach, intervention,* and *dentist*. Upon identification of relevant articles, the reference lists and any cited references were manually searched for further relevant literature. This search strategy was deemed to meet the PRESS Checklist for Elements for the Peer Review of Electronic Search Strategies [31] (Additional file 3).

### Study inclusion and exclusion criteria

All articles that were relevant to the study aims and published in the searched databases from inception up to 7 April 2019 were eligible for inclusion in this review. Inclusion and exclusion criteria have been presented in Table 1 according to the Population, Intervention, Control, Outcome (PICO) framework. Included studies followed experimental or quasi-experimental designs, including randomised controlled trials, pretest-posttest studies, interrupted time series studies and non-equivalent groups studies. Observational studies such as cross-sectional surveys, case-control studies and cohort studies were excluded from this review.

**Table 1:** Inclusion and exclusion criteria according to the PICO framework

Population	
Inclusion criteria	⦁ Participants with a qualification in any dental profession. This could include dentists, specialists in endodontics, periodontics, orthodontics and special needs dentistry, dental assistants, dental therapists and dental hygienists ⦁ Participants that practiced in a clinical dental care setting
Exclusion criteria	⦁ Participants that follow oral- or dental-related guidelines but are not a dental practitioner, for example an ear, nose and throat surgeon or a nurse providing oral care
Intervention	
Inclusion criteria	⦁ Any strategy that was utilised to facilitate the implementation of clinical guidelines into practice. These could include single interventions, which utilise a sole strategy, such as audit and feedback, education or reminders, and multifaceted interventions, which utilise multiple strategies concurrently
Exclusion criteria	⦁ Involved guideline dissemination as part of the intervention, meaning the comparison group or participants at baseline would not be aware of the guidelines to be able to implement them into practice
Control	
Inclusion criteria	⦁ Exposure to disseminated guidelines only. Thus, participants in control groups should be aware of the existence of the guidelines, but no further intervention should be provided to facilitate their uptake
Exclusion criteria	⦁ No exposure to disseminated guidelines
Outcome	
Inclusion criteria	⦁ Focussed on guideline adherence as a primary outcome. This could be measured by count or percentage of instances of guideline-adherent behaviour over a set time period. This could be performed prospectively using observation or retrospectively using audit or other similar methods
Exclusion criteria	⦁ Focussed on other outcome measures such as patient outcomes instead of guideline adherence

### Terminology

There are numerous health professionals that provide dental care to individuals. Among the most well-known are dentists, who can practice in general dentistry, or go on to specialise in various areas, including endodontics, periodontics, orthodontics and special needs dentistry [32–34]. However other dental practitioners work as part of the dental team to provide direct care to individuals, including dental assistants, dental therapists and dental hygienists [33, 35, 36]. In this review, the term *dental practice* was used to encompass all of the above professions, and *dental practitioner* encompassed the care providers working in these professions. Previous systematic reviews have commonly categorised implementation strategies into *single interventions*, which utilise a sole strategy, such as audit and feedback, education or reminders, and *multifaceted interventions*, which utilise multiple strategies concurrently [16, 17]. These terms were also used to classify implementation strategies in this review.

### Study quality assessment

The risk of bias and quality of each study was assessed using the Joanna Briggs Institute critical appraisal tools for both randomised controlled trials and quasi-experimental designs [37, 38] (Tables 2 and 3). This was initially performed by the first author and then independently reviewed by a second author (LR or DM). In the instance of any discrepancies in assessment, a third author (LR or DM) was involved to achieve consensus. This third author was not able to identify who provided each assessment. The appraisal tools were used to develop a score for each article, as a percentage of the number of met criteria out of the total number of applicable criteria. Through consensus among the authors, cut-off values were established prior to scoring, whereby studies with a score of less than 30% were excluded from analysis, 30–59% were considered poor quality, 60–79% were considered moderate quality and greater than 80% were considered high quality [54].

**Table 2:** Summaries of included studies

First author, year, country	Aims	Study design	Study population	EPOC categories [intervention(s)]	Comparator group	Outcome
Afuakwah, C., 2015, Scotland	Improve documentation of caries risk assessments (CRA)	Pretest-posttest quasi-experimental study	Four dentists working at a general dental practice in a Scottish Index of Multiple Deprivation One area	Multifaceted intervention: ⦁ Reminders [CRA pro forma, aide memoire] ⦁ Education (NFS) [staff training]	N/A	Adherence improved from 52.5% pre-intervention to 100% post-intervention
Amemori, M., 2013, Finland	Develop and evaluate two interventions intended to increase the implementation of tobacco use prevention and cessation counselling	Cluster randomised controlled trial	75 dentists and dental hygienists employed at 34 clinics within two municipal health care regions in Finland	⦁ Education (meetings) [lectures, interactive sessions, multimedia demonstrations and role play session (*n* = 21)] ⦁ Multifaceted intervention (*n* = 27): o Education (meetings) [as above] o Pay for performance [fee for service]	No intervention (*n* = 25)	⦁ No effect on prevention counselling for any group ⦁ Cessation counselling 6 months post-intervention was higher for intervention groups (effect size = 0.52, *p* = 0.007), despite a relapse after 2 months
Bahrami, M., 2004, Scotland	Evaluate the effectiveness of different implementation strategies for clinical guidelines relating to the management of impacted and unerupted third molar teeth	Pragmatic, 2 × 2 factorial cluster randomised controlled trial	51 general dental practices in Scotland who had been given the opportunity to attend a postgraduate course regarding the guidelines	⦁ Reminders [computer-aided learning with decision support (*n* = 13)] ⦁ Audit and feedback [audit and feedback (*n* = 13)] ⦁ Multifaceted intervention (*n* = 13): o Reminders [as above] ⦁ Audit and feedback [as above]	No intervention (*n* = 12)	No significant difference in guideline adherence was seen between intervention and control groups
Chopra, R., 2014, UK	To audit dentists’ antimicrobial prescription and evaluate the effectiveness of education on their adherence to antimicrobial prescribing guidelines	Pretest-posttest quasi-experimental study	Two audit cycles each including 60 patients in the dental department of a hospital in London	Education (meetings) [extensive training and education of staff and students]	N/A	A 50% increase in appropriate prescriptions was seen post intervention, as was a 38% increase in practitioners recording a diagnosis
Elouafkaoui, P., 2016, Scotland	Compare the impact of individualised audit and feedback interventions on dentists’ antibiotic prescribing rates	Cluster randomised controlled trial	2566 dentists from 795 general dental practices	⦁ Audit and feedback [audit and feedback (*n* = 1999)]	Current practice (*n* = 567)	⦁ A 5.7% greater decrease in antibiotic prescription (*p* = 0.01) was seen among the intervention groups ⦁ Defined daily dose rate reduced by 6.6% more in the intervention group (*p* = 0.03)
Friction, J., 2011, USA	Compare the impact of two reminder approaches on access of guidelines for patients with medically complex conditions	Randomised clinical trial	109 dentists from 15 dental clinics	⦁ Reminders [computer alerts to providers (*n* = 32)] ⦁ Patient-mediated interventions [notifications to patients (*n* = 38)]	Usual care (*n* = 39)	Both interventions increased guideline website use by 19% for the first 6 months (*p* < 0.05); however, this was not sustained to 12 months
Gnich, W., 2018, Scotland	Explore the effect of a financial incentive on frequency of fluoride varnish application(FVA) and underlying mechanisms	Non-equivalent groups quasi-experimental study	709 dentists who had submitted payment claims for dental services to the NHS primary care dental contract	Pay for performance [novel fee-for-service (*n* = 343)]	Continuous fee-for-service (*n* = 350)	FVA rates increased among both groups; however, a greater increase was seen among the intervention group (*β* = 0.82, 95% CI = 0.72–0.92)
Isaacson Tilliss, T., 2006, USA	To determine the effect of a multifaceted implementation strategy on oral cancer screening examinations and discussions of tobacco use	Cluster randomised controlled trial	31 dental care providers at 6 dental practices in Colorado	Multifaceted intervention (*n* = 18): ⦁ Local consensus process [local consensus process] ⦁ Reminders [multi-modal reminders for practitioners] ⦁ Patient-mediated interventions [multi-modal reminders for patients] ⦁ Education (meetings) [interactive educational workshop]	Usual care (*n* = 12)	No significant change was seen in patient reports of dental provider practice following the intervention, except a 22.1% (*p* = 0.015) increase in reporting “the dentist/hygienist told me that I was being screened for oral cancer”
Montini T., 2013, USA	To test the feasibility of using web-based computer-mediated clinical decision support system to improve dentists’ adherence to the Treating Tobacco Use and Dependence Clinical Practice Guidelines	Pretest-posttest quasi-experimental study	One general dental clinic located at the New York College of Dentistry	Reminders [computer decision support system]	N/A	⦁ Screening patients for tobacco use increased by 33.1% (*p* < 0.001) ⦁ Rates of advising, referring and prescribing nicotine replacement therapy for tobacco users increased by 58.9% (*p* < 0.001), 15.2% (*p* < 0.001) and 14.3% (*p* = 0.035) respectively
Rindal, D. B., 2013, USA	To determine the effect of a computer-assisted tobacco intervention tool on frequency of dentists’ adherence to tobacco guidelines	Cluster randomised controlled trial	548 patients from 15 HealthPartners Dental Group clinics in metropolitan Minnesota	Reminders [practitioners provided with computer decision support system]	Usual care	Rates of assessing interest in quitting (17%, *p* = 0.0006), discussing strategies (21%, *p* = 0.003) and referral (20%, *p* = 0.007) were significantly higher in the intervention group
Rosseel, J. P., 2012, The Netherlands	To examine the effect of patient-mediated feedback on adherence of dental practitioners to tobacco cessation guidelines	Pretest-posttest quasi-experimental study	23 primary care dental practices in the Netherlands, their professional personnel and patients	Patient-mediated interventions [patient-mediated feedback]	N/A	More patients reported receiving assessment of smoking status (25.3% increase, *p* < 0.01), information on smoking (21.3% increase, *p* < 0.01) and advice and support (26.5%, *p* < 0.01) 12 months post-intervention despite a 6.1% drop in reported provision of advice after 6 months
Shelley, D., 2011, USA	To evaluate the effect of a multicomponent intervention to implement tobacco use treatment guidelines in public health dental clinics	Pretest-posttest quasi-experimental study	14 comprehensive care general dentistry clinics at the New York College of Dentistry	Multifaceted intervention: ⦁ Reminders [chart system] ⦁ Education (meetings) [faculty and student training] ⦁ Environment [nicotine replacement therapy] ⦁ Referral systems [referral protocol] ⦁ Audit and feedback [referral feedback]	N/A	⦁ No significant difference in rates of screening for tobacco use ⦁ Rates of advising, assessing and referring or prescribing nicotine replacement therapy for tobacco users increased by 20.6% (*p* < 0.001), 12.1% (*p* = 0.01) and 9.1% (*p* = 0.01) respectively
Simons, D., 2013, UK	To determine the effects of an audit on the process and outcomes of clinical endodontic care	Pretest-posttest quasi-experimental study	20 clinicians within the Community Dental Service of the National Health Service	Audit and feedback [audit and feedback]	N/A	In general, there was increased adherence to various endodontic guidelines (0.7–42.9% increase), although this was not seen in all guidelines
Walsh, M. M., 2006, USA	To compare the effects of workshop training and mailed self-study training with and without reimbursement on tobacco-use-related attitudes and behaviours as reported by dentists and patients	Cluster randomised controlled trial with a 2 × 2 factorial design	265 dentists who participated in Delta Dental plans serving state employees in California, Pennsylvania and West Virginia	⦁ Education (materials) [self-study (*n* = 100)] ⦁ Education (meetings) [workshop (*n* = 99)]	No intervention (*n* = 66)	Although patient and self-reported adherence to tobacco guidelines was higher among both intervention groups, more dentists in the workshop group reported adherence than in the self-study group. Due to a low claim rate, reimbursement had no further effect on this
Zahabiyoun, S., 2015, UK	To determine whether clinical audit can improve use of antibiotics in the dental service	Pretest-posttest quasi-experimental study	Two dental clinics in the northeast of England	Audit and feedback [clinical audit]	N/A	⦁ Compliance with metronidazole prescription guidelines increased by 15.3% (*p* = 0.012) ⦁ Compliance with amoxicillin prescription guidelines increased by 35.2% (*p* = 0.041)

**Table 3:** Effectiveness of each implementation strategy

Type of implementation strategy	Type of outcome	Reported effects
Audit and feedback	Antibiotic guideline adherence	Significant improvement: [39, 40]
	Endodontic guideline adherence	Some improvement^†^: [41]
	Third molar guideline adherence	No improvement: [42]
Reminders	Tobacco cessation guideline adherence	Significant improvement: [43, 44]
	Medically complex conditions guideline adherence	Significant improvement, not sustained: [45]
	Third molar guideline adherence	No improvement: [42]
Education	Tobacco cessation guideline adherence	Significant improvement: [46] Some improvement: [47]
	Antibiotic guideline adherence	Improvement^†^: [48]
Patient-mediated interventions	Tobacco cessation guideline adherence	Significant improvement: [49]
	Medically complex conditions guideline adherence	Significant improvement, not sustained: [45]
Pay for performance	Fluoride varnish guideline adherence	Significant improvement: [50]
Multifaceted interventions	Tobacco cessation guideline adherence	Some improvement: [47, 51]
	Caries risk assessment guideline adherence	Improvement^†^: [52]
	Oral cancer screening guideline adherence	No improvement: [53]
	Third molar guideline adherence	No improvement: [42]

^†^No significance testing conducted

### Screening

A systematic screening process was conducted whereby initial search results were screened for eligibility first by title, then by abstract, and finally by full text. Titles and abstracts of identified records were screened independently by two authors (AV and DM), and a third author (AG) was invited to achieve consensus when discrepancies arose. For records that were not excluded by title or abstract, full texts were obtained and independently screened by the same investigators (AV and DM), and consensus was achieved by a third investigator (AG) where required.

### Data extraction strategy

Data from included studies was extracted by the first author using a data extraction tool (Additional file 4), which was validated through consensus with the research team, and included fields such as author, year, location, aims, study design, population and eligibility criteria, intervention/implementation strategies used and outcomes, measured by change in proportion of guideline-adherent practice where possible.

### Data synthesis and presentation

Due to high heterogeneity of included interventions, as well as the small number of articles meeting the eligibility criteria within each type of intervention, it was decided that a narrative synthesis of study findings would be undertaken. Studies were categorised by type of implementation strategy according to the Effective Practice and Organisation of Care (EPOC) taxonomy of health systems interventions [55]. This taxonomy was systematically and iteratively developed by the Cochrane EPOC Review Group to allow the classification of health systems interventions into categories based on conceptual or practical similarities [56]. Throughout the development process of this taxonomy, it was applied to various reviews of health systems interventions of high relevance to developing countries, making this a tool that could be relevant to a variety of settings [56]. In contrast, many other taxonomies, such as the ERIC taxonomy, only included panellists from specific geographic locations in their development, which could limit their applicability to studies conducted elsewhere [57]. Thus, the EPOC taxonomy was deemed an appropriate tool for the classification of the interventions used in this review. A process was adopted whereby initial classification was conducted by the first author (AV) who examined the interventions described within each study allocated these interventions to the EPOC category with the best matching definition. This categorisation was peer checked by other authors (LR, DM and AG). In the case where interventions consisted of components from multiple EPOC categories, these were classified as multifaceted interventions. All data was presented as a qualitative review.

### Registration

This systematic review was registered with the International Prospective Register of Systematic Reviews (PROSPERO), registration ID CRD42018093023.

## Results

### Search results

**Fig. 1:**
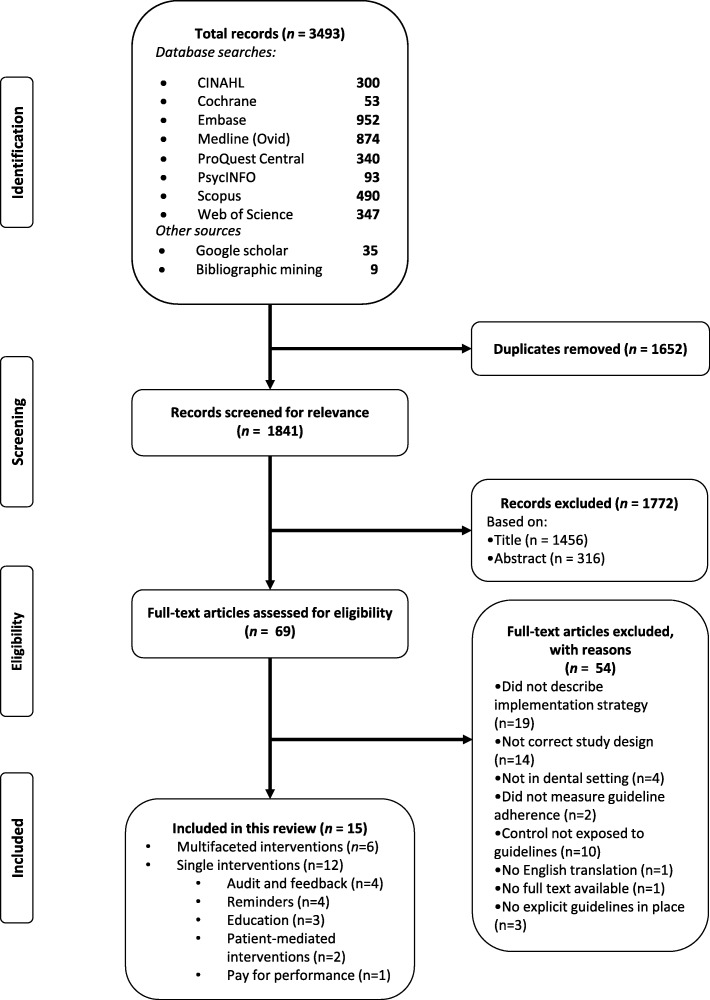
PRISMA flow chart for the systematic review

All database searches returned results, yielding a total of 3493 records. Following the removal of 1652 duplicates, titles and abstracts of records were screened, resulting in the exclusion of a further 1772 articles. Of the 96 full-text articles assessed, reasons for exclusion included the incorrect intervention (*n* = 19), incorrect study design (*n* = 14), not in the dental setting (*n* = 4), incorrect outcome (*n* = 2), the control group was not exposed to the guidelines (*n* = 10), no English full text available (*n* = 1) and no formal guidelines were in place (*n* = 3). A total of 15 studies were included in this review [39–53]. See Fig. 1 for further detail regarding the search and screening process.

### Study characteristics

Of the included studies, seven studies were from the UK [39–42, 48, 50, 52], six studies were from the USA [43–46, 51, 53], one study was from Finland [47] and one study was from the Netherlands [49]. Included studies were published between 2004 and 2018. A total of seven included studies were randomised controlled trials (RCTs) [39, 42, 44–47, 53], of which six were cluster randomised [39, 42, 44, 46, 47, 53] and one was individually randomised [45]. The remaining eight studies followed quasi-experimental designs, with seven being pretest-posttest quasi-experimental studies [40, 41, 43, 48, 49, 51, 52] and one being a non-equivalent groups design [50]. Out of all 15 studies, six involved implementation of interdisciplinary guidelines related to tobacco cessation into the dental setting [43, 44, 46, 47, 49, 51], and the rest explored the implementation of general dental guidelines [39–42, 45, 48, 50, 52, 53]. Implementation strategies employed in each study were classified into the following categories: audit and feedback (*n* = 4) [39–42], reminders (*n* = 4) [42–45], education (*n* = 3) [46–48], patient-mediated interventions (*n* = 2) [45, 49], pay for performance (*n* = 1) [50] and multifaceted interventions (*n* = 5) [42, 47, 51–53]. Three studies [42, 45, 47] were classified into multiple categories, as they included both multifaceted and single intervention arms. Details of the included studies can be seen in Table 2, and a summary of the effectiveness of implementation strategies in each category can be seen in Table 3.

### Quality assessment

The quality assessments of included studies are shown in Tables 4 and 5. All 15 studies received an acceptable quality assessment (score of 30% or higher) for inclusion in the review. Of the seven included RCTs, two were assessed to be of high quality [45, 46], four were of moderate quality [39, 42, 44, 47] and one was of poor quality [53] (Table 4). On average, RCTs had a score of 72.1%. The most common areas of methodological weakness in included RCTs were lack of blinding of both participants and those administering the intervention. This was expected due to the nature of the interventions making blinding difficult and unfeasible at times. Despite this, one RCT was able to implement a study design that permitted blinding [46]. Three of the eight included quasi-experimental studies was of high quality [49–51], with four being of moderate quality [40, 41, 43, 52], and one being of poor quality [48] (Table 5). Quasi-experimental studies had an average score of 74.08%. Weaknesses among these studies included limited use of control groups and lack of reliability of measures.

**Table 4:** Quality assessment of included randomised controlled trials

	Study identification						
Criteria	[47]	[42]	[39]	[45]	[53]	[44]	[46]
1. Was true randomisation used for assignment of participants to treatment groups?	Y	Y	Y	Y	?	Y	Y
2. Was allocation to treatment groups concealed?	Y	Y	Y	Y	N	?	Y
3. Were treatment groups similar at the baseline?	N	Y	N	Y	Y	Y	Y
4. Were participants blind to treatment assignment?	N	N	N	N	N	?	Y
5. Were those delivering treatment blind to treatment assignment?	N	N	N	N	N	N	Y
6. Were outcomes assessors blind to treatment assignment?	Y	Y	Y	Y	N	N	Y
7. Were treatment groups treated identically other than the intervention of interest?	Y	Y	Y	Y	Y	Y	Y
8. Was follow-up complete and if not, were differences between groups in terms of their follow up adequately described and analysed?	Y	Y	Y	Y	Y	Y	Y
9. Were participants analysed in the groups to which they were randomised?	Y	Y	Y	Y	Y	Y	Y
10. Were outcomes measured in the same way for treatment groups?	Y	Y	Y	Y	Y	Y	Y
11. Were outcomes measured in a reliable way?	Y	Y	Y	Y	Y	Y	Y
12. Was appropriate statistical analysis used?	Y	Y	Y	Y	N	Y	Y
13. Was the trial design appropriate, and any deviations from the standard RCT design (individual randomisation, parallel groups) accounted for in the conduct and analysis of the trial?	Y	Y	Y	Y	N	Y	Y
Total score	76.9%	76.9%	69.2%	92.3%	46.2%	69.2%	100.0%

*Y* yes, *N* no, ? unclear, *N/A* not applicable

**Table 5:** Quality assessment of included quasi-experimental studies

	Study identification							
Criteria	[52]	[48]	[50]	[43]	[49]	[51]	[41]	[40]
1. Is it clear in the study what is the “cause” and what is the “effect” (i.e. there is no confusion about which variable comes first)?	Y	Y	Y	Y	Y	Y	Y	Y
2. Were the participants included in any comparisons similar?	Y	?	Y	?	Y	Y	Y	Y
3. Were the participants included in any comparisons receiving similar treatment/care, other than the exposure or intervention of interest?	Y	Y	Y	Y	Y	Y	?	Y
4. Was there a control group?	N	N	Y	N	N	N	N	N
5. Were there multiple measurements of the outcome both pre and post the intervention/exposure?	Y	Y	Y	Y	Y	Y	Y	Y
6. Was follow-up complete and if not, were differences between groups in terms of their follow-up adequately described and analysed?	Y	N/A	Y	N/A	Y	N/A	N/A	N/A
7. Were the outcomes of participants included in any comparisons measured in the same way?	Y	Y	Y	Y	Y	Y	Y	Y
8. Were outcomes measured in a reliable way?	?	?	N	N	Y	N/A	Y	N
9. Was appropriate statistical analysis used?	N	?	Y	Y	Y	Y	Y	Y
Total score	66.7%	50.0%	88.9%	62.5%	88.9%	85.7%	75.0%	75.0%

*Y* yes, *N* no, ? unclear, *N/A* not applicable

### Single interventions

The 12 studies classified into single intervention categories involved interventions classified as audit and feedback (*n* = 4) [39–42], reminders (*n* = 4) [42–45], education (*n* = 3) [46–48], patient-mediated interventions (*n* = 2) [45, 49] and pay for performance (*n* = 1) [50].

#### Audit and feedback

Four studies explored the effectiveness of audit and feedback interventions on the implementation of guidelines in dental practice [39–42]. This category included two cluster RCTs [39, 42] and three pretest-posttest quasi-experimental studies [40, 41, 49]. The study by Zahaboyoun et al. found audit and feedback to significantly increase compliance with both metronidazole and amoxicillin prescription guidelines [40]. Similarly, the study by Elouafkaoui et al. found a significant decrease in antibiotic prescription following an audit and feedback intervention [39]. Simons and Williams reported mixed results, where audit and feedback increased adherence to some, but not all, endodontic guidelines; however, the statistical significance of these changes was not evaluated [41]. Conversely, the cluster RCT by Bahrami et al. [42] showed no significant improvement in guideline adherence to unerupted third molar guidelines following an audit and feedback intervention.

#### Reminders

An additional four studies investigated the effect of reminder strategies on guideline implementation [42–45]. Of these studies, three were RCTs [42, 44, 45] and one was a quasi-experimental study [43]. The quasi-experimental study by Montini et al. found that a computer decision support system significantly improved not only tobacco screening rates, but also rates of advising, referring and prescribing nicotine replacement therapy for tobacco users [43]. Similarly, the computer decision support system used by Rindal et al. also significantly improved clinicians’ adherence to tobacco cessation guidelines [44]. However, while increasing guideline adherence for the first 6 months, the study by Friction et al. found that computer alerts to providers did not cause sustained change in adherence to guidelines for patients with medically complex conditions [45]. In addition, the computer-aided learning and decision support strategy used by Bahrami et al. showed no significant effect on guideline adherence [42].

#### Education

Three studies evaluated the effectiveness of education strategies on improving guideline adherence [46–48], two of which were RCTs [46, 47]. The cluster RCT by Walsh et al. found that although both self-study and workshop-based education strategies improved self-reported adherence to tobacco cessation guidelines, there was higher reported adherence in the workshop intervention [46]. In addition, the pretest-posttest quasi-experimental study by Chopra et al. found that extensive training and education caused an increase in adherence to antimicrobial prescribing guidelines, despite having no statistical evaluation of this effect [48]. However, Amemori et al. found mixed results, concluding that an education package consisting of lectures, interactive sessions, multimedia demonstrations and a role play session resulted in a significant increase in provision of tobacco cessation counselling, but not tobacco prevention counselling [47].

#### Patient-mediated interventions

Two studies explored the use of patient-mediated interventions as guideline implementation strategies. Rosseel et al. found that patient-mediated feedback increased the proportion of patients reporting having received guideline-adherent information, advice and support regarding tobacco cessation; however, this declined after 6 months [49]. In addition, the study by Friction et al. found that notifying patients to ask for a review of care during their visit caused an increase in adherence to guidelines for patients with medically complex conditions in the first 6 months; however, this was not sustained [45].

#### Pay for performance

Finally, one non-equivalent groups quasi-experimental study by Gnich et al. evaluated the effectiveness of fee-for-service as a single intervention to improve guideline implementation [50]. The investigators found that guideline-adherent fluoride varnish application rates increased rapidly among practitioners that had received fee-for-service during the intervention, when compared to practitioners who had already been receiving fee-for-service prior to the intervention.

### Multifaceted interventions

A total of five studies tested the effectiveness of a multifaceted intervention and were therefore included in this category [42, 47, 51–53]. This category included three RCT studies [42, 47, 53], with the remaining two studies following pretest-posttest quasi-experimental study designs [51, 52]. The multifaceted interventions utilised in these studies varied and comprised of a combination of two or more implementation strategies including education, audit and feedback, fee-for-service or decision support (see Table 2). All five studies in this category involved education in combination with other strategies as part of the multifaceted intervention. Four studies combined education with reminders [42, 51–53], two utilised audit and feedback [42, 51], and one employed a pay for performance strategy [47].

Although results seen within each study varied, three studies [47, 51, 53] showed a significant increase in some component of guideline implementation following the multifaceted intervention. These studies [47, 51, 53], which explored the implementation of multicomponent tobacco cessation guidelines, highlighted mixed results, with multifaceted interventions causing a significant improvement in adherence to some guideline components, but no change was seen in other components. Similar to their single intervention, Amemori et al. [47] highlighted that their education plus fee-for-service intervention caused a significant increase in tobacco cessation counselling; however, no change was seen in tobacco prevention counselling. Similarly, the multicomponent intervention utilised by Shelley et al. [51], which involved a chart system, training, protocols and referral feedback, resulted in a significant increase in providing advice, assessments and assistance to tobacco users; however, rates of tobacco use screening remained the same. Finally, the local consensus process, multi-modal reminders for patients and practitioners and interactive educational workshop utilised by Isaacson et al. caused no significant change in patient-reported adherence to oral cancer screening guidelines aside from patient agreement with the statement “the dentist/hygienist told me that I was being screened for oral cancer” [53].

The multifaceted interventions in the remaining two studies [42, 52] did not exhibit significant changes in adherence for various reasons. One study by Afuakwah and Welbury [52] indicated that a pro forma, aide memoire and staff training had a positive effect on adherence to guidelines regarding documentation of caries risk assessments. However, this study did not compute any inferential statistics; therefore, the significance of this change could not be ascertained. The study by Bahrami et al. did compute inferential statistics to determine the effect of a multifaceted intervention involving computer-aided learning with decision support plus audit and feedback on adherence; however, no significant improvement was detected.

**Fig. 2:**
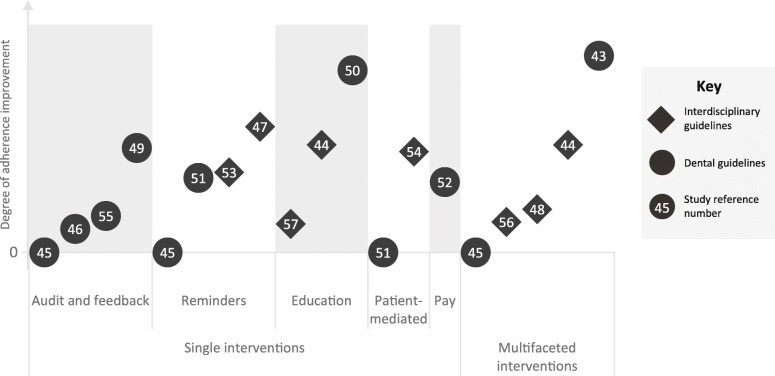
A diagrammatic representation of the effect of each study intervention on improvement in adherence, grouped by intervention category and guideline type

As seen in Fig. 2, overall, a slightly higher proportion of studies involving multifaceted interventions reported improvements in guideline adherence when compared to single interventions. Among single interventions, studies classified into the “reminders” and “education” categories reported larger improvements in guideline adherence. In addition, all studies involving interdisciplinary guidelines reported some effect of the implementation strategies, on guideline adherence, whereas over 20% of studies involving dental guidelines reported no effect.

## Discussion

With existing research highlighting dental practice to have some of the lowest guideline compliance in the health sector, it is evident that the implementation of clinical guidelines in this setting remains a challenge [18–21]. As no published reviews summarise the evidence regarding implementation strategies in the dental setting, this review aimed to evaluate the current research in this area and identify effective guideline implementation strategies. This review identified a total of 15 studies that investigated the effectiveness of implementation strategies in the dental setting, with 13 studies investigating single implementation strategies and 5 studies involving multifaceted implementation strategies. There were studies classified across the categories of audit and feedback [39–41], reminders [43, 44], education [46–48], patient-mediated interventions [49], pay for performance [50] and multifaceted interventions [47, 51–53] that reported a significant increase in adherence to some or all guideline components.

Within the single interventions, the effectiveness of audit and feedback interventions was variable, with the three successful studies showing slightly smaller effect sizes than studies in other categories [39–41]. Systematic reviews of implementation strategies in other health care settings have also reported audit and feedback interventions to have variable outcomes on guideline adherence [17, 58]. Ivers et al. [58] reported that audit and feedback interventions may be more effective when baseline performance is low; when the source of feedback is a supervisor or colleague; when they are provided more than once; when they are delivered in both verbal and written formats; and when they include both explicit targets and action plans. Hypotheses developed by Colquhoun et al have reinforced these findings, also suggesting that involvement of recipients in the design of the audit and feedback process could improve the effectiveness of these interventions [59].

Three of four studies within this review using reminders reported guideline adherence to improve following intervention [43–45], with higher improvements indicating reminders may be a more effective strategy in the dental setting than audit and feedback. Other reviews have also highlighted reminders as a promising strategy to improve guideline adherence [17, 60–63], and one review has even highlighted the potential for this strategy to positively impact on patient outcomes [64]. It is suggested that computerised decision support systems may be more effective in improving compliance; however, they may also lengthen consultation times and may be more stressful for clinicians to use [17]. Further, studies evaluating reminder systems developed specifically by the organisations where implementation will occur have shown larger effects on improving practice than the adoption of existing reminder systems [62].

Similar to other health care settings [17, 60, 61], this review found education to be an effective implementation strategy, with three of four included studies in this category reporting improvement in guideline adherence [46–48]. However, the intervention consisting of passive education strategies, that is, in the “educational materials” EPOC category, resulted in lower effects when compared with other interventions [46]. This is consistent with other literature, which highlight passive education strategies to be largely unsuccessful in improving guideline adherence [17].

Only one out of the two studies in the patient-reported feedback demonstrated a significant improvement in guideline adherence, indicating some potential variability in the effectiveness of this implementation strategy; however, more research is required to confirm this [49]. Reviews in other health care settings have shown these types of interventions to be more effective [17, 65].

Although the sole study investigating a pay-for-service intervention in this review reported a moderate significant effect on guideline adherence [50], interestingly, this type of implementation strategy was less often explored in other settings with inconclusive results found regarding its effectiveness [17, 60, 61]. The effectiveness of this strategy was hypothesised to be due to its ability to normalise or validate the responsibility of dental care practitioners in performing the service.

Of the five studies exploring multifaceted interventions in this review, four highlighted positive effects on guideline adherence [47, 51–53]. Previous studies in other settings have shown huge success of multifaceted interventions, showing them to be much more effective than single implementation strategies alone, making them one of the most frequently researched and recommended approaches for guideline implementation [17, 61]. Despite this, there is little evidence regarding the best components to include in these strategies, as there does not seem to be a relationship between number or type of components used and effectiveness of the strategy [17].

Despite this evidence of positive effects across all types of implementation strategies, some studies only found partial effects on implementation, and further there were some studies that did not show evidence of effect on guideline adherence. Many of the studies that showed only partial effects had some characteristics in common, the first of which was complexity of guidelines. All five studies that only found partial improvements in guideline adherence involved complex guidelines with multiple criteria for compliance, often combined with interdisciplinary practice [41, 46, 47, 51, 53]. This is supported by previous research in other settings, with previous reviews highlighting guideline complexity as the single most frequently cited guideline characteristic affecting guideline implementation [66].

Further, the two studies that highlighted no effect of their implementation strategies on guideline adherence had some methodological limitations that may have impacted on the effects of the implementation strategies. Firstly, Bahrami et al. acknowledged a high baseline compliance in their study, which may have produced a ceiling effect, meaning that no greater improvement was possible following the intervention [42]. In addition, this is the oldest study included in this review, conducted in 2004, yet it heavily involved the use of computer decision support systems as part of the implementation strategies, despite the fact that computer systems were not commonly used in patient care at that time. This is reinforced by the fact that the authors specifically developed the computer software package for the purpose of this study, contained on a stand-alone laptop [42]. As a result, clinicians would be required to specifically use this laptop when decision support was required, rather than the decision support being embedded into computer patient records as was commonly done in more recent studies [43, 44], potentially reducing compliance to this decision support intervention. The second study that did not find overall significant improvements was Friction et al., which used the frequency of accessing an online computer decision support tool as an indicator of guideline adherence [45]. The limitation of this proxy, acknowledged by the authors, was that there was the potential that the more the decision support tool was used, the more clinicians may begin to learn the guidelines, and as a result may not have needed to refer to the decision support repeatedly [45]. This is reinforced by the trend in the data that increases in guideline adherence were seen at 6 months, but regressed to baseline at 12 months [45].

In summary, this study has highlighted that implementation strategies such as audit and feedback, reminders, education, patient-mediated interventions, pay for performance and multifaceted interventions have all had some success in the dental setting, with reminders, education, pay for performance and multifaceted interventions showing the most promise. Further research is required to provide more high-quality evidence regarding the effectiveness of each strategy type and gain an understanding of aspects of each type of strategy that may increase the success of guideline implementation. Despite some promising findings in this review, it has several limitations that should be considered in the interpretation of results. Firstly, the quality of evidence identified by this review varied, with most of the 15 included studies being of poor or moderate quality, and only five studies being deemed high quality. In addition, two included studies did not conduct statistical tests for the positive changes they identified following their implementation strategies, one of which only involved four clinicians, further impacting the interpretability of the study findings [48, 52]. Further, due to the heterogeneity of this data, resulting from the variability of implementation strategies used in each study, meta-analysis was not possible in this review, and as a result, the effects of each strategy could not be quantitatively compared. A large number of included studies were published in the UK and USA, which could limit generalisability of findings. In addition, although rigorous search strategies were used, there is a chance that not all relevant studies were identified, and due to a paucity of research in this field, relatively few studies were ultimately included. Finally, this study excluded studies for which full texts or English translations could not be obtained, which may have potentially introduced some bias into the results of the review. Nonetheless, this study is the first systematic review of implementation strategies in the dental setting and has provided significant insight into which strategies may be most effective for the implementation of guidelines in this sector.

## Conclusions

This study has confirmed findings in other settings that implementation strategies such as audit and feedback, reminders, education, patient-mediated interventions and multifaceted interventions may be effective in improving guideline adherence in the dental setting. It has highlighted that interventions such as education, reminders and multifaceted interventions may be the most effective in this setting, and it has identified pay for performance as a potentially effective strategy that has previously been inconclusive in other settings. Although some included studies showed equivocal findings or no effects on guideline adherence, key strategies were identified that could be utilised in the implementation of any future dental guidelines, as well as considerations that should be taken into account in the use of these strategies. This information is particularly relevant in light of the increased need and focus on role expansion of dental professionals into other areas like childhood obesity. This review highlights the need for further, high-quality research to be conducted in this setting, to gain a better understanding of the conditions under which each strategy works best. Increasing the number of studies using rigorous methods within each strategy category will allow heterogeneity of findings to be reduced, therefore enabling meta-analyses to be conducted.

## Supplementary information

**Additional file 1.** PRISMA checklist.

**Additional file 2.** Search strategy.

**Additional file 3.** PRESS checklist.

**Additional file 4.** Data extraction tool.

## Data Availability

Not applicable

## Abbreviations

CRA: Caries risk assessment
EPOC: Effective Practice and Organisation of Care
FVA: Fluoride varnish application
MeSH: Medical Subject Headings
PRISMA: Preferred reporting items for systematic reviews and meta-analyses
PROSPERO: International Prospective Register of Systematic Reviews
RCT: Randomised controlled trial

## References

[1] Institute of Medicine (US) Committee to Advise the Public Health Service on Clinical Practice Guidelines (1990). Clinical practice guidelines: directions for a new program.

[2] Grimshaw JM, Russell IT (1993). Effect of clinical guidelines on medical practice: a systematic review of rigorous evaluations. Lancet..

[3] Woolf SH (1993). Practice guidelines: a new reality in medicine: iii. impact on patient care. Archives of Internal Medicine..

[4] Woolf SH, Grol R, Hutchinson A, Eccles M, Grimshaw J (1999). Potential benefits, limitations, and harms of clinical guidelines. British Medical Journal (Clinical Research Ed)..

[5] Proctor EK, Landsverk J, Aarons G, Chambers D, Glisson C, Mittman B (2009). Implementation research in mental health services: an emerging science with conceptual, methodological, and training challenges. Administration and Policy in Mental Health and Mental Health Services Research..

[6] US Department of Health and Human Services (2006). The road ahead: research partnerships to transform services. A report by the National Advisory Mental Health Council’s Workgroup on Services and Clinical Epidemiology Research.

[7] Bernhardsson S, Johansson K, Nilsen P, Öberg B, Larsson MEH (2014). Determinants of guideline use in primary care physical therapy: a cross-sectional survey of attitudes, knowledge, and behavior. Physical Therapy..

[8] Flottorp SA, Oxman AD, Krause J, Musila NR, Wensing M, Godycki-Cwirko M (2013). A checklist for identifying determinants of practice: a systematic review and synthesis of frameworks and taxonomies of factors that prevent or enable improvements in healthcare professional practice. Implementation Science..

[9] Hsiao J-L, Chen R-F (2016). Critical factors influencing physicians’ intention to use computerized clinical practice guidelines: an integrative model of activity theory and the technology acceptance model. BMC Medical Informatics and Decision Making..

[10] Jones JA, Reeve CA (2018). Factors influencing the use of clinical guidelines by general practitioners working in a setting of complex multimorbidity: a case study by interviews. BMC Fam Pract.

[11] Sauro KM, Wiebe S, Holroyd-Leduc J, DeCoster C, Quan H, Bell M (2018). Knowledge translation of clinical practice guidelines among neurologists: a mixed-methods study. PLoS One.

[12] Ouimet M, Landry R, Amara N, Belkhodja O (2006). What factors induce health care decision-makers to use clinical guidelines? Evidence from provincial health ministries, regional health authorities and hospitals in Canada. Social Science & Medicine..

[13] Conroy M, Shannon W (1995). Clinical guidelines: their implementation in general practice. British journal of general practice..

[14] Cabana MD, Rand CS, Powe NR (1999). Why don’t physicians follow clinical practice guidelines? A framework for improvement. JAMA..

[15] Grol R, Grimshaw J (2003). From best evidence to best practice: effective implementation of change in patients’ care. Lancet..

[16] Grimshaw J, Thomas R, MacLennan G, Fraser C, Ramsay C, Vale L (2004). Effectiveness and efficiency of guideline dissemination and implementation strategies.

[17] Prior M, Guerin M, Grimmer-Somers K (2008). The effectiveness of clinical guideline implementation strategies – a synthesis of systematic review findings. Journal of Evaluation in Clinical Practice..

[18] Grilli R, Lomas J (1994). Evaluating the message: the relationship between compliance rate and the subject of a practice guideline. Medical care..

[19] Cleveland JL, Foster M, Barker L, Gordon Brown G, Lenfestey N, Lux L (2012). Advancing infection control in dental care settings: factors associated with dentists’ implementation of guidelines from the Centers for Disease Control and Prevention. Journal of the American Dental Association..

[20] Spallek H, Song M, Polk DE, Bekhuis T, Frantsve-Hawley J, Aravamudhan K (2010). Barriers to implementing evidence-based clinical guidelines: a survey of early adopters. Journal of Evidence-Based Dental Practice..

[21] Polk DE, Weyant RJ, Shah NH, Fellows JL, Pihlstrom DJ, Frantsve-Hawley J. Barriers to sealant guideline implementation within a multi-site managed care dental practice. BMC Oral Health. 2018;18(1). 10.1186/s12903-018-0480-z PMC5797385 29394921

[22] Gordon JS, Lichtenstein E, Severson HH, Andrews JA (2006). Tobacco cessation in dental settings: research findings and future directions. Drug and Alcohol Review..

[23] Lalla E, Kunzel C, Burkett S, Cheng B, Lamster IB (2011). Identification of unrecognized diabetes and pre-diabetes in a dental setting. Journal of Dental Research..

[24] Tseng R, Vann WF, Perrin EM (2010). Addressing childhood overweight and obesity in the dental office: rationale and practical guidelines. Pediatric Dentistry..

[25] Khan KS, Kunz R, Kleijnen J, Antes G (2003). Five steps to conducting a systematic review. J R Soc Med..

[26] Moher D, Liberati A, Tetzlaff J, Altman DG, The PG (2009). Preferred reporting items for systematic reviews and meta-analyses: the PRISMA statement. PLOS Medicine..

[27] Kong AC, Ramjan L, Sousa MS, Gwynne K, Goulding J, Jones N, et al. The oral health of Indigenous pregnant women: a mixed-methods systematic review. Women and Birth. 2019. 10.1016/j.wombi.2019.08.007 31501053

[28] Ramjan L, Cotton A, Algoso M, Peters K (2016). Barriers to breast and cervical cancer screening for women with physical disability: a review. Women Health..

[29] George A, Johnson M, Blinkhorn A, Ellis S, Bhole S, Ajwani S (2010). Promoting oral health during pregnancy: current evidence and implications for Australian midwives. J Clin Nurs..

[30] Villarosa AR, George D, Ramjan LM, Srinivas R, George A (2018). The role of dental practitioners in addressing overweight and obesity among children: a scoping review of current interventions and strategies. Obes Res Clin Pract..

[31] McGowan J, Sampson M, Lefebvre C (2010). An evidence based checklist for the peer review of electronic search strategies (PRESS EBC). Evidence Based Library and Information Practice.

[32] American Dental Association. General dentistry 2019 [Available from: https://www.ada.org/en/education-careers/careers-in-dentistry/general-dentistry.

[33] British Dental Association. BDA advice: careers in dentistry 2011 [Available from: https://bda.org/dcps/working-in-dentistry/Documents/e12_careers_in_dentistry_-_sept_11.pdf#search=dental%2520careers.

[34] Australian Dental Association. Dental specialists 2019 [Available from: https://www.ada.org.au/Careers/Specialists.

[35] American Dental Association. Dental team careers 2019 [Available from: https://www.ada.org/en/education-careers/careers-in-dentistry/dental-team-careers.

[36] Australian Dental Association. Dental team 2019 [Available from: https://www.ada.org.au/Careers/Dental-Team.

[37] The Joanna Briggs Institute. The Joanna Briggs Institute critical appraisal tools for use in JBI systematic reviews: checklist for randomized controlled trials 2017 [Available from: http://joannabriggs.org/assets/docs/critical-appraisal-tools/JBI_RCTs_Appraisal_tool2017.pdf.

[38] The Joanna Briggs Institute. The Joanna Briggs Institute critical appraisal tools for use in JBI systematic reviews: checklist for quasi-experimental studies (non-randomized experimental studies) 2017 [Available from: http://joannabriggs.org/assets/docs/critical-appraisal-tools/JBI_Quasi-Experimental_Appraisal_Tool2017.pdf.

[39] Elouafkaoui P, Young L, Newlands R, Duncan EM, Elders A, Clarkson JE (2016). An audit and feedback intervention for reducing antibiotic prescribing in general dental practice: the RAPiD cluster randomised controlled trial. Plos Medicine.

[40] Zahabiyoun S, Sahabi M, Kharazi MJ (2015). Improving knowledge of general dental practitioners on antibiotic prescribing by raising awareness of the faculty of general dental practice (UK) guidelines. Journal of Dentistry (Tehran, Iran).

[41] Simons D., Williams D. (2013). Can audit improve patient care and treatment outcomes in endodontics?. British Dental Journal.

[42] Bahrami M, Deery C, Clarkson JE, Pitts NB, Johnston M, Ricketts I (2004). Effectiveness of strategies to disseminate and implement clinical guidelines for the management of impacted and unerupted third molars in primary dental care, a cluster randomised controlled trial. British Dental Journal..

[43] Montini T, Schenkel AB, Shelley DR (2013). Feasibility of a computerized clinical decision support system for treating tobacco use in dental clinics. Journal of Dental Education..

[44] Rindal DB, Rush WA, Schleyer TKL, Kirshner M, Boyle RG, Thoele MJ (2013). Computer-assisted guidance for dental office tobacco-cessation counseling: a randomized controlled trial. American Journal of Preventive Medicine..

[45] Fricton J, Rindal DB, Rush W, Flottemesch T, Vazquez G, Thoele MJ (2011). The effect of electronic health records on the use of clinical care guidelines for patients with medically complex conditions. Journal of the American Dental Association..

[46] Walsh MM, Belek M, Prakash P, Grimes B, Heckman B, Kaufman N (2012). The effect of training on the use of tobacco-use cessation guidelines in dental settings. Journal of the American Dental Association..

[47] Amemori M, Virtanen J, Korhonen T, Kinnunen TH, Murtomaa H (2013). Impact of educational intervention on implementation of tobacco counselling among oral health professionals: a cluster-randomized community trial. Community Dentistry and Oral Epidemiology..

[48] Chopra R, Merali R, Paolinelis G, Kwok J (2014). An audit of antimicrobial prescribing in an acute dental care department. Primary dental journal..

[49] Rosseel JP, Jacobs JE, Hilberink SR, Segaar D, Akkermans R, Maassen IM (2012). Patient-reported feedback promotes delivery of smoking cessation advice by dental professionals. International Journal of Health Promotion and Education..

[50] Gnich W, Sherriff A, Bonetti D, Conway DI, Macpherson LMD. The effect of introducing a financial incentive to promote application of fluoride varnish in dental practice in Scotland: a natural experiment. Implementation science: IS. 2018;13(1). 10.1186/s13012-018-0775-0 PMC6042272 29996868

[51] Shelley D, Anno J, Tseng TY, Calip G, Wedeles J, Lloyd M (2011). Implementing tobacco use treatment guidelines in public health dental clinics in New York City. Journal of Dental Education..

[52] Afuakwah Charles, Welbury Richard (2015). Why Do You Need to Use a Caries Risk Assessment Protocol to Provide an Effective Caries Preventive Regime?. Primary Dental Journal.

[53] Isaacson TT (2006). Improving implementation of oral cancer screening recommendations [Ph.D.].

[54] Goldsmith MR, Bankhead CR, Austoker J (2007). Synthesising quantitative and qualitative research in evidence-based patient information. Journal of epidemiology and community health..

[55] Effective Practice and Organisation of Care. EPOC taxonomy 2015 [Available from: https://epoc.cochrane.org/epoc-taxonomy.

[56] Effective Practice and Organisation of Care. The EPOC taxonomy of health systems interventions Oslo: Norwegian Knowledge Centre for Health Services; 2016 [Available from: https://epoc.cochrane.org/resources/epoc-resources-review-authors.

[57] Powell BJ, Waltz TJ, Chinman MJ, Damschroder LJ, Smith JL, Matthieu MM (2015). A refined compilation of implementation strategies: results from the Expert Recommendations for Implementing Change (ERIC) project. Implementation Science..

[58] Ivers N, Jamtvedt G, Flottorp S, Young JM, Odgaard-Jensen J, French SD, et al. Audit and feedback: effects on professional practice and healthcare outcomes. Cochrane Database Syst Rev. 2012;6. 10.1002/14651858.CD000259.pub3 PMC11338587 22696318

[59] Colquhoun HL, Carroll K, Eva KW, Grimshaw JM, Ivers N, Michie S (2017). Advancing the literature on designing audit and feedback interventions: identifying theory-informed hypotheses. Implementation Science..

[60] Ebben RHA, Siqeca F, Madsen UR, Vloet LCM, van Achterberg T (2018). Effectiveness of implementation strategies for the improvement of guideline and protocol adherence in emergency care: a systematic review. BMJ Open..

[61] Watkins K, Wood H, Schneider CR, Clifford R (2015). Effectiveness of implementation strategies for clinical guidelines to community pharmacy: a systematic review. Implementation science : IS..

[62] Ammenwerth E, Schnell-Inderst P, Machan C, Siebert U (2008). The effect of electronic prescribing on medication errors and adverse drug events: a systematic review. J Am Med Inform Assoc..

[63] Cheung A, Weir M, Mayhew A, Kozloff N, Brown K, Grimshaw J (2012). Overview of systematic reviews of the effectiveness of reminders in improving healthcare professional behavior. Systematic Reviews..

[64] Durieux P, Trinquart L, Colombet I, Nies J, Walton R, Rajeswaran A (2008). Computerized advice on drug dosage to improve prescribing practice. Cochrane Database Syst Rev.

[65] Fonhus MS, Dalsbo TK, Johansen M, Fretheim A, Skirbekk H, Flottorp SA (2018). Patient-mediated interventions to improve professional practice. Cochrane Database Syst Rev.

[66] Francke AL, Smit MC, de Veer AJ, Mistiaen P (2008). Factors influencing the implementation of clinical guidelines for health care professionals: a systematic meta-review. BMC Medical Informatics and Decision Making..

