# Supplementary material for: The effectiveness of guideline implementation strategies in the dental setting: a systematic review
Source: Implement Sci. 2019 Dec 17;14:106. doi: 10.1186/s13012-019-0954-7 (PMC6918615; doi:10.1186/s13012-019-0954-7)
Supplement: Supplementary file 2 — Additional file 2. Search strategy. [file 13012_2019_954_MOESM2_ESM.docx]

## Additional file 1: Search strategy

| Database | Search | Results |
| --- | --- | --- |
| Scopus | ( TITLE-ABS-KEY ( guideline* OR recommendation* OR consensus ) AND TITLE-ABS-KEY ( implement* OR disseminat* OR translat* ) AND TITLE-ABS-KEY ( strateg* OR approach* OR intervention* ) AND TITLE-ABS-KEY ( dental OR dentist* ) ) | 485 |
| Scopus | Secondary documents from above search (found in reference lists) | 5 |
| CINAHL | ( guideline* OR recommendation* OR consensus ) AND ( implement* OR disseminat* OR translat* ) AND ( trategy* OR approach* OR intervention* ) AND ( dental OR dentist* ) | 162 |
| CINAHL | (MH “Dentists”) AND ((MH “Guideline Adherence”) OR (MH “Practice Guidelines”)) | 138 |
| Medline | ( guideline* OR recommendation* OR consensus ) AND ( implement* OR disseminat* OR translat* ) AND ( strateg* OR approach* OR intervention* ) AND ( dental OR dentist* ) | 786 |
| Medline | (MH "Guideline Adherence") AND ((MH "Dentists") OR (MH “Dentistry”) | 88 |
| ProQuest | ( guideline* OR recommendation* OR consensus ) AND ( implement* OR disseminat* OR translat* ) AND ( strateg* OR approach* OR intervention* ) AND ( dental OR dentist* ) | 322 |
| ProQuest | MESH(Guideline Adherence) AND MESH(Dentists) | 18 |
| PsycINFO | ( guideline* OR recommendation* OR consensus ) AND ( implement* OR disseminat* OR translat* ) AND ( strateg* OR approach* OR intervention* ) AND ( dental OR dentist* ) | 92 |
| PsycINFO | (DE "Dentists") AND (DE "Treatment Guidelines") | 1 |
| Embase | ((guideline* or recommendation* or consensus) and (implement* or disseminat* or translat*) and (strateg* or approach* or intervention*) and (dental or dentist*)) | 510 |
| Embase | (practice guideline/ AND dentist/) | 442 |
| Web of Science | TOPIC: (guideline* OR recommendation* OR consensus) AND TOPIC: (implement* OR disseminat* OR translat*) AND TOPIC: (strateg* OR approach* OR intervention*) AND TOPIC: (dental OR dentist*) | 347 |
| Cochrane | guideline* or recommendation* or consensus in Title Abstract Keyword AND implement* or disseminat* or translat* in Title Abstract Keyword AND strateg* or approach* or intervention* in Title Abstract Keyword AND dental or dentist* in Title Abstract Keyword - (Word variations have been searched) | 51 |
| Cochrane | (MeSH descriptor: [Guideline Adherence] explode all trees) AND (MeSH descriptor: [Dentists] explode all trees) | 2 |
| Scholar | guideline implementation strategy dental OR dentist | 35 |
