# Supplementary material for: The effectiveness of guideline implementation strategies in the dental setting: a systematic review
Source: Implement Sci. 2019 Dec 17;14:106. doi: 10.1186/s13012-019-0954-7 (PMC6918615; doi:10.1186/s13012-019-0954-7)
Supplement: Supplementary file 3 — Additional file 3. PRESS checklist. [file 13012_2019_954_MOESM3_ESM.docx]

## Additional file 3: PRESS checklist

| **Translation of the research question** | Has the research question been translated correctly into search concepts (e.g. PICO), i.e. does the search strategy match the research question? | Yes |
| --- | --- | --- |
|  | Are the search concepts clear? | Yes |
|  | Are there ‘too many’ search concepts? | No |
|  | Are any of the search concepts too narrow or too broad? | No |
|  | Does the search appear to retrieve too many or too few records? | No |
| **Boolean and proximity operators** | Are there any mistakes in the use of Boolean or proximity operators? | No |
|  | Are there any mistakes in the use of nesting with brackets? | No |
|  | If NOT is used, is this likely to result in any unintended exclusions? | N/A |
|  | Could precision be improved by using proximity operators (e.g. adjacent, near, within) instead of AND? | No |
|  | Is the width of any proximity operators correct? | N/A |
| **Subject headings** | Are the subject headings relevant? | Yes |
|  | Are subject headings missing? | No |
|  | Are any subject headings too broad or too narrow? | No |
|  | Are subject headings exploded where necessary and vice versa? | Yes |
|  | Are sub-headings attached to subject headings? (Floating subheadings may be preferred) | Yes |
|  | Are sub-headings used instead of relevant subject headings and vice versa? | No |
|  | Are both subject headings and natural language terms used for each concept?   - If there is a reason provided for not doing so, does the reason appear sound? | Yes |
| **Natural language (also free-text or text-word)** | Does the search miss any spelling variants in free-text? | No |
|  | Does the search miss any synonyms? | No |
|  | Does the search miss truncation or truncate at the wrong point? | No |
|  | If an acronym or abbreviation is used, is the full term also included? | N/A |
|  | Are apparently irrelevant or excessively broad natural language terms used | No |
| **Spelling, syntax and line numbers** | Are there any spelling errors? | No |
|  | Are there any errors in the system syntax or wrong line numbers? | No |
| **Limits and filters** | Do any of the limits used seem unwarranted? | No |
|  | Are any filters used appropriate for the topic? | N/A |
|  | Are any potentially helpful limits or filters missing? | No |
|  | Is starring (restrict to focus) used and if so, is there adequate justification for this? | No |
| **Search strategy adaptations** | Does the searcher indicate that the search strategy has been adapted for additional databases and/or interfaces | Yes |
|  | Are the adaptations available for review and correct? | Yes |
