# Supplementary material for: The effectiveness of guideline implementation strategies in the dental setting: a systematic review
Source: Implement Sci. 2019 Dec 17;14:106. doi: 10.1186/s13012-019-0954-7 (PMC6918615; doi:10.1186/s13012-019-0954-7)
Supplement: Supplementary file 4 — Additional file 4. Data extraction tool. [file 13012_2019_954_MOESM4_ESM.docx]

## Additional file 4: Data extraction tool

| **First Author/ Year/ Country** | **Aims** | **Study Design** | **Study Population** | **Intervention(s)** | **Comparison** | **Outcome** | **Critical Appraisal** |
| --- | --- | --- | --- | --- | --- | --- | --- |
|  |  |  |  |  |  |  |  |
|  |  |  |  |  |  |  |  |
|  |  |  |  |  |  |  |  |
|  |  |  |  |  |  |  |  |
|  |  |  |  |  |  |  |  |
|  |  |  |  |  |  |  |  |
|  |  |  |  |  |  |  |  |
|  |  |  |  |  |  |  |  |
|  |  |  |  |  |  |  |  |
|  |  |  |  |  |  |  |  |
|  |  |  |  |  |  |  |  |
|  |  |  |  |  |  |  |  |
|  |  |  |  |  |  |  |  |
|  |  |  |  |  |  |  |  |
|  |  |  |  |  |  |  |  |
|  |  |  |  |  |  |  |  |
